# The Identification of a Glucuronyltransferase-Related Gene, *GlcAT-S,* with Putative Mucus Protection and Anti-Inflammatory Effects from Gut-Damaged *Drosophila* by Dextran Sulfate Sodium (DSS)

**DOI:** 10.3390/biology14050513

**Published:** 2025-05-07

**Authors:** Seung Hun Lee, Dooseon Hwang, Jang-Won Lee, Tae-Won Goo, Eun-Young Yun

**Affiliations:** 1 Department of Integrative Biological Sciences and Industry, Sejong University, Seoul 05006, Republic of Korea; g-d-lsh@hanmail.net (S.H.L.); h.michael8837@gmail.com (D.H.); wintrelove@sejong.ac.kr (J.-W.L.); 2 Department of Future Food and Resources Technology, Donga University of Health, Yeongam 58439, Republic of Korea; 3 Department of Biochemistry, School of Medicine, Dongguk University, Gyeongju 38766, Republic of Korea

**Keywords:** *Drosophila* gut-damage model, bioinformatics tools, inflammation, *GlcAT-S*, mucin

## Abstract

The intestinal epithelium, which is protected by mucosal surfaces composed of mucins and other glycoproteins, functions as a selective barrier that absorbs nutrients while preventing the translocation of harmful substances. To understand the mechanisms between mucosal disruption and tissue inflammation, we orally administrated a mucus-disrupting agent, dextran sodium sulfate, to *Drosophila melanogaster* and screened 63 differentially expressed genes (DEGs). Through a database search using bioinformatics tools (CHEA3 and WebGestalt), we identified ELK1 as a potential key transcription factor for the selected DEGs, and among the 63 DEGs, ELK1-related genes, B3GAT3, FIBP, and TENT2 (*GlcAT-S*, *Fibp*, and *Wisp* in *Drosophila*), were selected as the relevant genes that respond to mucus disruption. We confirmed that enterocyte (EC)-specific *GlcAT-S* knockdown by RNAi significantly reduced gut length and increased intestinal stem cell proliferation in *Drosophila*. Additionally, in EC-specific *GlcAT-S*-knockdown flies, it was observed that the mucus-production-related genes, *Muc68D* and *Mur29B*, were specifically reduced, whereas the inflammatory cytokines *egr* and *upd3* were overexpressed. This study provides evidence that *GlcAT-S* is involved in the regulation of intestinal inflammation in *Drosophila* and plays a protective role against mucus disruption. Our findings suggest that *GlcAT-S* may be a potential therapeutic target for the treatment of intestinal inflammatory diseases such as IBD.

## Simple Summary

The present study provides evidence that *GlcAT-S* plays a protective role in the maintenance of gut tissue integrity and the regulation of intestinal inflammation in a *Drosophila* gut-damage model. The disruption of the mucosal barrier is a key feature of inflammatory bowel disease, and understanding the mechanisms underlying this disruption and the subsequent inflammatory response is crucial for developing effective therapies. Using a combination of bioinformatics analysis and genetic manipulation, we identified *GlcAT-S* as a key gene that is involved in mucosal protection, as well as demonstrating its role in DSS-induced intestinal damage. Our findings suggest that *GlcAT-S* may serve as a potential therapeutic target for the treatment of inflammatory bowel disease.

## 1. Introduction

IBD is a chronic, multifactorial condition that is characterized by inflammation of the tissue and increased permeability [1]. Disruption of the mucosal barrier is a hallmark of IBD and is closely associated with disease progression and pathogenesis [2]. The intestinal mucus layer plays a crucial role in maintaining gut homeostasis and protecting against tissue damage [3]. In conditions such as ulcerative colitis, alterations in the mucus composition, thickness, or continuity can lead to increased bacterial penetration and subsequent inflammation [3]. However, IBD is caused by various factors such as organ failure, chemical exposure, microbiota disruption, stress, unhealthy diet, and surgery [4]. Therefore, identifying key genes involved in gut tissue inflammation following mucosal disruption could be essential in understanding the mechanism of the complex disease.

The *Drosophila melanogaster* is a powerful model organism for studying a wide range of human diseases, due to its similarity to human-disease-related genes [5]. Especially, *Drosophila* gut has a similar tissue structure, cellular components, and gene response to that in mammals; therefore, it is used as a gut disease model [6]. The *Drosophila* midgut shares molecular and cellular homology with mammalian intestines, making it a robust model for studying gut regeneration, barrier function, and inflammation [6]. Equivalent to the mammalian gut mucus layer, a peritrophic matrix (PM), acting as a physical defense, is present in the *Drosophila* intestine [7]. PM is a highly organized layer that is composed of glycosaminoglycan (GAG) and chitin [8], which are synthesized by multiple genes and enzymes such as glucuronyltransferases. The oral administration of dextran sulfate sodium (DSS) is widely used to induce gut inflammation by disrupting the mucosal barrier; this model provides insights into the mechanisms of intestinal inflammation and potential therapeutic targets for IBD treatment [9]. The DSS is the most widely used to achieve IBD murine models [10] and is also used to induce the *Drosophila* IBD model [11]. The *Drosophila* intestinal epithelium possesses a complex protective system, featuring both a semi-permeable PM of chitin and proteins and a thin mucus layer, detectable by periodic acid–Schiff (PAS) staining [12,13]. Thus, the oral-administration of DSS to *Drosophila* is a reliable IBD model. Moreover, *Drosophila* is amenable to genetic manipulation, allowing for the generation of transgenic flies that express specific genes or RNAi constructs [14]. This feature makes *Drosophila* an ideal model for studying the role of specific genes in the pathogenesis of IBD. However, the exact mechanism of GAGs in the mucus-disrupted model remains incompletely understood.

RNA sequencing provides a very large number of gene expression data; thus, the characterization of differentially expressed genes (DEGs) through bioinformatics tools is a widely used method due to its efficiency [15]. Bioinformatics tools have played a crucial role in discovering key genes in certain multifactorial diseases such as inflammatory bowel disease (IBD) [16,17], aiding in the screening of core genes from large genetic datasets [18].

In this study, we used bioinformatics tools to screen DEGs from the sequencing data of a DSS-induced *Drosophila* gut-damage model and investigated the role of *GlcAT-S* in intestinal inflammation in this model. We generated enterocyte (EC)-specific *GlcAT-S*-mutant flies and assessed their susceptibility to DSS-induced intestinal inflammation. Our results demonstrate that *GlcAT-S*-mutant flies are more susceptible to DSS-induced intestinal inflammation, suggesting that *GlcAT-S* plays a protective role against gut tissue inflammation. This study provides the first evidence that *GlcAT-S* is involved in the regulation of intestinal inflammation in *Drosophila*. Our findings suggest that *GlcAT-S* may be a potential therapeutic target for the treatment of IBD.

## 2. Materials and Methods

### 2.1. Experimental Drosophila and Feeding Assay

*Drosophila melanogaster* was reared on a standard cornmeal–yeast–sucrose diet at 18 °C until the initiation of feeding experiments. Adult flies were collected at a 1:1 male-to-female ratio using light CO_2_ anesthesia and maintained under controlled conditions (25 °C, 65% relative humidity, 12 h light/dark cycle) until use in experiments. For feeding assays, groups of 50 flies (25 males and 25 females) were housed in vials containing chromatography paper (2.5 cm × 3.75 cm) soaked with 500 μL of the specified test solutions. The control group received 5% sucrose, while the experimental group was exposed to a different treatment: 5% sucrose with 7% dextran sulfate sodium (DSS, MW 40 kDa; MP Biomedicals, Solon, OH, USA) to induce gut damage. Feeding experiments were conducted at 29 °C. The vials and media-soaked papers were replaced daily, and the mortality was recorded throughout this study.

### 2.2. cDNA Synthesis and Quantitative PCR Analysis

*Drosophila* midguts were dissected and homogenized, followed by total RNA extraction using TRIzol reagent (Ambion, Carlsbad, CA, USA) according to the manufacturer’s protocol. The extracted RNA was further purified using lithium chloride precipitation solution (Invitrogen, Carlsbad, CA, USA) in order to enhance purity and integrity. Complementary DNA (cDNA) was synthesized from 1 μg of total RNA using the QuantiNova Reverse Transcription Kit (QIAGEN GmbH, Hilden, Germany), following the manufacturer’s instructions. A quantitative polymerase chain reaction (qPCR) was conducted using the QuantiNova SYBR Green PCR Kit (QIAGEN GmbH, Hilden, Germany) on a CFX96 thermocycler (Bio-Rad Laboratories, Hercules, CA, USA).

### 2.3. EC-Specific Knockdown of Selected Genes Using RNA Interference

We specifically knocked down gene expression in ECs using the Myo31DF-Gal4 driver line [19], in conjunction with a temperature-sensitive tubGal80^ts^ driver line (#67067, Bloomington *Drosophila* stock center (BDSC), Bloomington, IN, USA) [20]. Temperature-sensitive tubGal80^ts^ suppresses Gal4 at 18 °C by expressing Gal80, while tubGal80^ts^ is inactivated at 29 °C, thus inducing Gal4 expression. Three RNAi lines were obtained from the BDSC: GlcAT-S (#67781), Fibp (#63665), and Wisp (#43141). Male UAS-RNAi transgenic flies were crossed with virgin driver line females at a 1:3 ratio. The resulting F1 progeny and all crosses were reared at 18 °C until adulthood, and 3- to 5-day-old adults were subsequently shifted to 29 °C for 2 days to induce RNAi expression by inhibiting Gal80.

### 2.4. Drosophila Gut Immunostaining and Imaging

*Drosophila* midguts were dissected in ice-cold 4% paraformaldehyde (PFA) and fixed at room temperature for either 20 min or 60 min, depending on the experimental conditions. Following fixation, the samples were washed three times with phosphate-buffered saline (PBS) (10 min each), followed by two washes in PBS containing 0.1% Triton X-100 (Duksan reagents, Ansan, Republic of Korea) (PBST) (30 min each). To reduce non-specific binding, the samples were blocked for 90 min in a blocking buffer (PBST supplemented with 3% bovine serum albumin). For phospho-histone 3 (PH3) staining, the samples were incubated overnight at 4 °C with primary antibody (rabbit anti-PH3, 1:600, Cell Signaling Technology, Beverly, MA, USA) diluted in blocking buffer. The following day, the samples were washed four times in PBSTA (PBST containing 0.5% bovine serum albumin) (20 min each) and incubated with secondary antibody (goat anti-rabbit IgG Alexa Fluor 568, 1:2000, Invitrogen, Rockford, IL, USA) for 180 min at room temperature. After secondary antibody incubation, the samples were washed three times in PBST (20 min each), briefly rinsed in PBS three times, and mounted on slides using VECTASHIELD PLUS mounting medium containing DAPI (Vector Laboratory, Burlingame, CA, USA). For imaging, the samples were visualized using an Olympus BX51 fluorescence microscope (Olympus Optical Co., Tokyo, Japan) equipped with a JNOPTIC BKONV KON3.1 CMOS camera (JNOPTIC Co., Seoul, Republic of Korea). Female *Drosophila* gut lengths were measured using JNO-ARM software (version 3.7.401; JNOPTIC Co., Seoul, Republic of Korea) by tracing segmented curves along the center of each midgut (the midgut region starts from the proventriculus boundary to the midgut–hindgut junction, identified by morphology observation). For each group, five guts were initially measured, resulting in *n* = 5. The intestinal stem cells (ISCs) were quantified by counting the PH3-positive cells in the entire midgut. The imaging parameters were standardized across all the samples, and the cell counts were averaged from five different regions per experimental group.

### 2.5. Statistical Analysis

All the statistical analyses were performed using Student’s *t*-test in GraphPad Prism 6 (GraphPad Software Inc., San Diego, CA, USA), with significance defined as *p* < 0.05.

## 3. Results

### 3.1. Identification of Key DEGs from the DSS-Induced Drosophila IBD Model Through Multiple Bioinformatics Tools

**Figure 1:**
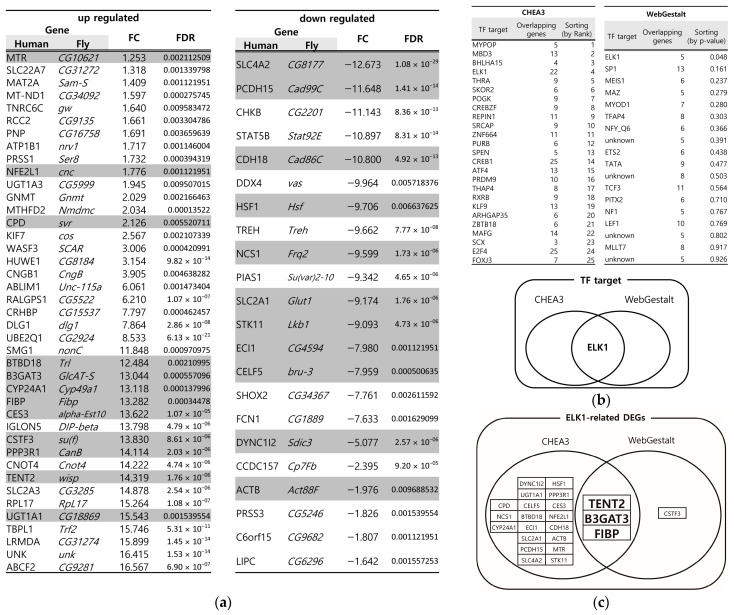
Essential gene screening of *Drosophila* mucus disruption model by bioinformatics tools. (**a**) Differentially expressed genes (DEGs) were identified from the sequencing data of DSS-administered *Drosophila*, with ortholog conversion via Isobase. In total, 41 genes were upregulated, and 22 genes were downregulated. The DEGs were selected by a certain significance level (Log fold change (FC) ≥ |1|, false detection rate (FDR) < 0.01). The highlighted DEGs are ELK-1 related. (**b**) The total DEGs were then screened using two different transcription factor target databases, CHEA3 and WebGestalt, revealing ELK1 as a common transcription factor target. (**c**) Further analysis of the ELK1-related genes within our DEGs identified 22 genes from the CHEA3 database and 4 genes from the WebGestalt database. Notably, B3GAT3, FIBP, and TENT2 were identified as overlapping genes (*GlcAT-S*, *Fibp*, and *Wisp* in *Drosophila*).

In IBD, the disruption of the mucus layer is a common feature, leading to increased gut permeability, heightened immune responses, and chronic inflammation [4]. We previously identified DEGs in *Drosophila* following oral administration of the mucus-disrupting agent DSS [21]. First, we converted the *Drosophila* DEGs to their human orthologs by Isobase [22], resulting in 41 upregulated and 22 downregulated DEGs (Figure 1a). To account for incomplete network databases in bioinformatics tools, we used two different tools, CHEA3 (ChIP-X Enrichment Analysis) [23] and WebGestalt (WEB-based Gene Set Analysis Toolkit) [24], and screened for overlapping genes via transcription factor (TF) enrichment analysis to improve the accuracy of essential gene identification. Using these bioinformatics tools, ELK1 was the only overlapping TF identified (Figure 1b). Furthermore, from the selected DEGs (Figure 1a), we identified three genes (B3GAT3, Beta-1,3-Glucuronyltransferase 3; FIBP, FGF1 Intracellular Binding Protein; and TENT2, Terminal Nucleotidyltransferase 2) that are related to ELK1 as a TF (Figure 1c). B3GAT3 and its family (B3GAT2) are genes that encode an enzyme for the biosynthesis of GAGs that catalyze the glycosaminoglycan–protein linkage [25]. FIBP encodes the acidic fibroblast growth factor intracellular-binding protein, which plays several important roles such as mitogenesis, embryonic development, and colorectal cancer chemoresistance [26]. TENT2 is a cytoplasmic poly(A) RNA polymerase that plays crucial roles in RNA modification and regulation [27]. Since the B3GAT family is involved in GAG biosynthesis, it may be related to the PM and mucus. However, such associations have not yet been reported for the other genes.

### 3.2. EC-Specific Knockdown of Screened DEGs

**Figure 2:**
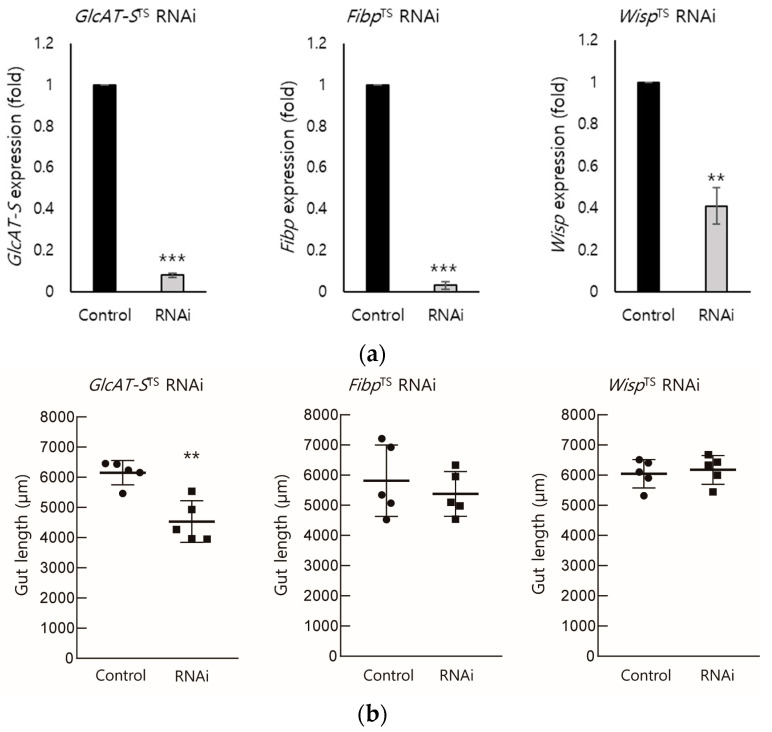
Enterocyte (EC)-specific knockdown of three screened DEGs. To investigate the roles of three genes (*GlcAT-S*, *Fibp*, and *Wisp*) in ECs, we performed EC-specific knockdown using RNA interference (RNAi) with a temperature-sensitive Myo31DF driver line. (**a**) After 2 days to induce RNAi expression, successful knockdown of each gene in the gut tissue was confirmed using qPCR. (**b**) The measurement of the gut length in the knockdown lines revealed a significant shortening of the gut, specifically in flies with EC-specific *GlcAT-S* knockdown. Error bars represent SD (*n* = 5, ** *p* < 0.01, *** *p* < 0.001, *t*-test).

To determine the role of *Drosophila* B3GAT3, FIBP, and TENT2 (*GlcAT-S*, *Fibp*, and *Wisp*) in the gut tissue following DSS-mediated injury, we generated EC-specific RNAi lines to knockdown the expression of these genes in ECs, the major cell type that forms the epithelium of the gut tissue [28,29]. We crossed enterocyte-specific driver line Myo31DF-Gal4 (w*; P{GawB}Myo31DF[NP0001]/CyO;P{UAS-3xFLAG.dCas9.VPR}attP2, P{tubP-GAL80[ts]}2), which express Gal4 in the Myo31DF expression pattern, and temperature-sensitive Gal80. The driver line was crossed with RNAi lines, which express dsRNA for the RNAi of each gene under UAS control. We then confirmed the transcriptional knockdown of each gene in the gut tissue of the respective RNAi lines using qRT-PCR (Figure 2a). Given that gut length reduction is an important indicator of tissue damage [30], we measured the gut length in the knockdown lines. Compared with the control group (6267.2 μm), only *GlcAT-S* RNAi flies exhibited a significant reduction in gut length (4345.1 μm). These results suggest that, among the three DEGs, *GlcAT-S* is the most critical for maintaining gut tissue.

### 3.3. Confirmation of the Relationship Between GlcAT-S and Gut Tissue Damage Through EC-Specific Knockdown

**Figure 3:**
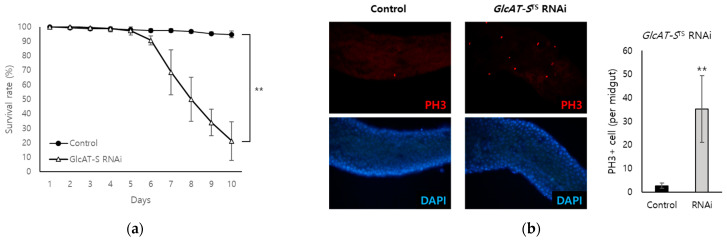
*Drosophila* EC-specific knockdown of *GlcAT-S* caused gut damage. To evaluate the roles of *GlcAT-S* in EC, EC-specific RNAi was induced by a temperature-sensitive Myo31DF driver line. (**a**) After 10 days, the survival rate of *Drosophila* (*n* = 50 per experiment, male:female = 1:1) was significantly reduced to 21.3% following *GlcAT-S* knockdown. Non-crossed driver lines were used as a control group. (**b**) Intestinal stem cell (ISC) proliferation was labeled by a phospho-histone H3 (PH3) mitotic marker. After 2 days to induce RNAi expression, ISC proliferation increased significantly to 35.3 cells (per gut) after EC-specific *GlcAT-S* knockdown, indicating gut damage and EC-derived cytokine release. Error bars represent SD (*n* = 3, ** *p* < 0.01, *t*-test compared with control group).

Increased numbers of *Drosophila* intestinal stem cells (ISCs) are a hallmark of EC damage, reflecting a tissue repair mechanism [31]. PH3 is a widely used marker for mitotic cells; in the *Drosophila* midgut, only ISCs proliferate and exhibit PH3 mitotic activity [32]. To confirm the importance of *GlcAT-S* in protecting against gut tissue damage, we quantified PH3-positive cells in the midgut of flies following EC-specific *GlcAT-S* knockdown. The EC-specific knockdown of *GlcAT-S* significantly reduced the survival rate of flies to 21.3% after 10 days, while the control group showed a 94.7% survival rate (Figure 3a). In addition, *GlcAT-S* knockdown dramatically increased the number of PH3+ cells per midgut, from 2.7 cells in control flies to 35.3 cells. Together, these data indicate that reduced EC-specific expression of *GlcAT-S* is associated with gut damage and leads to a decreased survival rate.

### 3.4. Analysis of the Relationship Among GlcAT-S, Mucus Loss, and Inflammation in the Drosophila Gut-Damage Model

**Figure 4:**
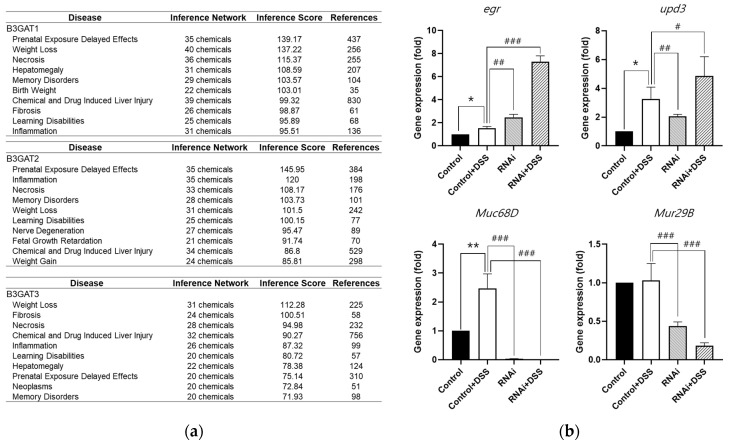
The inflammatory cytokine expression of EC-specific *GlcAT-S* RNAi *Drosophila* gut tissue. (**a**) *GlcAT-S* orthologs and related family members B3GAT1, B3GAT2, and B3GAT3 are strongly associated with human inflammation. The gene–disease relationship was confirmed from the Comparative Toxicogenomics Database (CTD) bioinformatics database. (**b**) The expression levels of two *Drosophila* cytokines, eiger (*egr*) and unpaired3 (*upd3*) as well as mucus-related genes *Muc68D* and *Mur29B*, were measured in the gut tissue. Error bars represent SD (*n* = 3, * *p* < 0.05, ** *p* < 0.01, # *p* < 0.05, ## *p* < 0.01, ### *p* < 0.001, *t*-test).

Inflammation is a complex biological response to harmful stimuli such as pathogens or damaged cells [33] and is a key component of the immune system’s defense mechanism, especially in gut tissue [34]. The mucus layer in the gut is a critical barrier that protects the underlying epithelial cells from damage [35]. Mucus loss is a common feature of IBD, and it can contribute to inflammation by allowing bacteria and other harmful substances to contact the epithelial cells [36]. The oral administration of DSS causes mucus loss in the gut tissue [37]. We hypothesized that altered *GlcAT-S* expression in DSS-induced *Drosophila* would be closely related to inflammation in ECs and to mucus formation covering the intestinal mucosa, as *GlcAT-S* is involved in the synthesis of various GAGs [38]. Through an analysis of the Comparative Toxicogenomics Database (CTD), we determined that the human ortholog genes, B3GAT1, B3GAT2, and B3GAT3 of *GlcAT-S* are associated with human inflammation (Figure 4a). Therefore, in this study, we analyzed whether *GlcAT-S* is related to inflammation not only in the human but also in the fly gut (Figure 4b). The EC-specific knockdown of *GlcAT-S* increased cytokineI expression in fly gut tissue. The *Drosophila* TNF ortholog, *eiger* (*egr*), and the IL-6 ortholog, *unpaird 3* (*upd3*), are important signaling molecules that act as cytokines. In *GlcAT-S* RNAi flies without DSS treatment, *egr* was upregulated by 2.47-fold and *upd3* was upregulated by 2.05-fold compared with the control group. In *GlcAT-S* RNAi flies administered orally with DSS, *egr* and *upd3* were induced to express up to 7.29- and 4.85-fold, respectively, which were higher than those in the *GlcAT-S*-knockdown group without DSS. These results suggest that increased inflammation in the gut tissue by the decreased expression of *GlcAT-S* in EC is further exacerbated by DSS. Intestinal epithelial cells constantly interact with their cellular environment, including components such as mucins and GAGs [39]. Both play crucial roles in maintaining the intestinal barrier function and mucosal immunity; the alteration of mucin [10] and GAG expression in the intestinal cell environment is associated with intestinal inflammation [40]. In *Drosophila*, Mucin 68D (*Muc68D*) and Mucin-related 29B (*Mur29B*) are the most specific mucin genes and are abundantly expressed in the fly intestine [41]. Given that *GlcAT-S* was identified from DSS-treated, mucus-disrupted flies, we examined *Muc68D* and *Mur29B* gene expression in the gut tissue. EC-specific knockdown of *GlcAT-S* specifically downregulated *Muc68D* to 0.03-fold and *Mur29B* to 0.44-fold, compared with the DSS-treated group (Figure 4b). These results indicate that *GlcAT-S* supports the mucus layer, preventing pathogen and harmful substance contact with epithelial cells. The loss of *GlcAT-S* disrupts this protective barrier, leading to increased cytokine production and reduced mucin gene expression. In short, these data indicate that *GlcAT-S* plays a protective role in *Drosophila* gut tissue.

## 4. Discussion

To identify the potential regulators of intestinal inflammation, we employed a multi-faceted approach integrating data from a DSS-induced *Drosophila* IBD model and bioinformatics tools (CHEA3 and WebGestalt). This in silico analysis enabled the identification of ELK1 as a potential key transcription factor influencing gut inflammation (Figure 1). CHEA3, or ChIP-X Enrichment Analysis, leverages ChIP-seq data and integrates orthogonal omics data to predict the TFs regulating a gene set. It focuses on direct TF–target interactions, analyzing binding site data from chromatin immunoprecipitation experiments, capturing a wider range of potential targets. In this study, CHEA3 identified 22 genes related to ELK1. WebGestalt, or WEB-based Gene Set Analysis Toolkit, uses functional annotations, which may require stronger statistical significance for gene inclusion, making it more restrictive. By overlapping the results, this study reduces false positives that might arise from incomplete or biased databases. CHEA3 offers a direct, binding-based view, while WebGestalt adds a functional context, ensuring a more complete understanding of TF regulation. Subsequently, we narrowed our focus to the ELK1-related genes: B3GAT3, FIBP, and TENT2 (corresponding to *GlcAT-S*, *Fibp*, and *Wisp* in *Drosophila*). An RNAi-based screening approach in *Drosophila* then revealed *GlcAT-S* as a potential regulator of intestinal inflammation (Figure 3 and Figure 4). The observed reduction in gut length in *GlcAT-S*-knockdown flies compared with that in controls suggests a critical role for *GlcAT-S* in maintaining gut tissue integrity (Figure 2). Gut shortening is a recognized hallmark of tissue damage and impaired epithelial renewal [30]. Our finding aligns with this, underscoring the importance of *GlcAT-S* in sustaining gut homeostasis under damaged conditions. The *GlcAT-S* gene encodes glucuronyltransferase S, an enzyme involved in the biosynthesis of glycosaminoglycans (GAGs) such as heparan sulfate and chondroitin sulfate [42]. GAGs are essential components of the extracellular matrix and play a key role in mucosal protection [43]. They contribute to the structure and function of proteoglycans, including those that interact with mucins to maintain the viscosity and stability of the mucus layer, which acts as a barrier protecting the underlying epithelial cells from damage [44,45]. Furthermore, previous studies have reported the upregulation of *GlcAT-S* by inflammatory cytokines, suggesting its potential involvement in the inflammatory response [46]. Elevated PH3 staining in *GlcAT-S*-knockdown flies (Figure 3) indicates an increase in ISC proliferation [32], further supporting the idea that *GlcAT-S* is essential for preventing gut damage. ISCs are responsible for regenerating damaged epithelial cells [47]; therefore, their increased proliferation suggests a response to tissue damage caused by the *GlcAT-S* deficiency. The reduced survival rate of *GlcAT-S*-knockdown flies further underscores that this gene is important not only for gut health but also for overall survival. These findings suggest that *GlcAT-S* plays a protective role in the gut, and its absence leads to increased susceptibility to tissue damage and reduced survival. The evolutionary conservation of GAG biosynthesis pathways suggests that *GlcAT-S* orthologs in humans, such as B3GAT1, B3GAT2, and B3GAT3, may play similar roles in intestinal homeostasis and inflammation [40]. Our bioinformatics analysis using the Comparative Toxicogenomics Database (CTD) supports this hypothesis (Figure 4a), as these human genes are highly associated with inflammation-related pathways. Importantly, *GlcAT-S* knockdown resulted in a significant upregulation of the inflammatory cytokines, *egr* and *upd3*, and the effect was amplified by DSS administration (Figure 4). This suggests that *GlcAT-S* deficiency not only predisposes the gut to damage but also promotes inflammatory responses in a mucus-disrupted environment. Additionally, we observed a significant downregulation of the key mucin genes *Muc68D* and *Mur29B* in EC-specific *GlcAT-S*-knockdown flies (Figure 4), given that mucins are major components of the mucus layer that protects the gut epithelium from damage [3,35,37,48]. Moreover, enterocytes can express several mucin genes in mammals [49] and flies [50,51]; this finding suggests that *GlcAT-S* in ECs is essential for maintaining mucus layer integrity. This connects *GlcAT-S* to the pathology of IBD, where mucus disruption plays a central role in disease progression.

The identification of *GlcAT-S* as a protective factor against intestinal inflammation has implications for understanding IBD pathogenesis. Our results suggest that *GlcAT-S* plays an important role in suppressing inflammatory responses. Given its role in GAG biosynthesis, alterations in GAG composition may be a contributing factor to IBD development. DSS treatment increases *Muc68D* and *Mur29B* expression as a compensatory response to gut damage, likely through inflammatory signaling pathways. However, in *GlcAT-S* RNAi flies, whether with or without DSS, these genes are downregulated compared with those in the DSS-treated control group, indicating that *GlcAT-S* is necessary for both the basal and induced expression of mucus genes. This suggests that the perturbation of *GlcAT-S* enzymatic activity disrupts the signaling environment required for transcription, possibly by reducing GAG-mediated signaling pathways by signaling molecules such as fibroblast growth factor (FGF), epidermal growth factor (EGF), and transforming growth factor-beta (TGF-β) or altering the extracellular matrix’s regulatory role. Furthermore, the increased susceptibility of *GlcAT-S*-knockdown flies to DSS-induced inflammation suggests that individuals with impaired *GlcAT-S* function may be at an increased risk for developing IBD or experiencing more severe disease progression. These findings could potentially be used to inform personalized medicine approaches in IBD treatment and prevention. These results suggest that the integrated strategy of in silico prediction followed by in vivo validation used in this study is effective in identifying novel regulators of gut homeostasis.

## 5. Conclusions

Our study highlights the protective role of *GlcAT-S* in mitigating intestinal inflammation and preserving gut tissue integrity in a *Drosophila* model of IBD. By demonstrating that *GlcAT-S* knockdown exacerbates gut damage, increases inflammatory cytokine expression, and disrupts mucus homeostasis, we provide novel insights into the molecular mechanisms underlying IBD pathogenesis. These findings indicate that further exploration of *GlcAT-S* and its human orthologs as potential therapeutic targets for the treatment of IBD and other gastrointestinal disorders is warranted. Future research should focus on elucidating the exact molecular interactions between *GlcAT-S* and inflammatory pathways and on exploring pharmacological strategies to modulate its activity in disease contexts.

## Data Availability

Data will be available upon request from the corresponding author.
